# Predicting Blood Loss and Evaluating Tranexamic Acid Efficacy in Staged Bilateral Total Knee Arthroplasty

**DOI:** 10.7759/cureus.82030

**Published:** 2025-04-10

**Authors:** Yoshinori Ishii, Hideo Noguchi, Junko Sato, Ikuko Takahashi, Hana Ishii, Ryo Ishii, Kai Ishii, Shin-ichi Toyabe

**Affiliations:** 1 Orthopaedic Surgery, Ishii Orthopaedic and Rehabilitation Clinic, Gyoda, JPN; 2 Orthopaedics, Ishii Orthopaedic and Rehabilitation Clinic, Gyoda, JPN; 3 Plastic and Reconstructive Surgery, Ishii Orthopaedic and Rehabilitation Clinic, Gyoda, JPN; 4 Orthopaedic Surgery, Shinshu University Hospital, Matsumoto, JPN; 5 Division of Information Science and Biostatistics, Niigata University Hospital, Niigata, JPN

**Keywords:** administration timing, blood loss, prediction, staged bilateral tka, total knee arthroplasty (tka), tranexamic acid (txa)

## Abstract

Background

This retrospective study aimed to evaluate the predictive capacity of first-stage blood loss for second-stage blood loss and to assess the impact of varying tranexamic acid (TXA) administration timing on hemostatic efficacy within the same patient undergoing staged bilateral total knee arthroplasty (TKA).

Methods

A retrospective analysis was conducted on 100 patients (200 knees) who underwent staged bilateral primary TKA performed by a single surgeon. Patients were categorized into three groups based on TXA administration: no TXA (NT, 106 knees), TXA administered only during the second stage (H, 42 knees), and TXA administered during both procedures (T, 52 knees).

Results

In the NT group, a moderate correlation was observed between blood loss in the first and second legs (p=0.042, r=0.438), suggesting that first-stage blood loss can predict second-stage blood loss. The H group showed a trend towards reduced blood loss in the second, TXA-treated leg (p=0.068), indicating a potential benefit of TXA administration in the second stage. However, no significant difference in blood loss was observed between the two legs in the T group (p=0.657), and no correlation was found between blood loss in the two legs (p=0.070).

Conclusions

These findings suggest that in non-TXA cases, first-stage blood loss can be used to tailor perioperative management for the second stage. Furthermore, the variability in TXA efficacy highlights the need for individualized TXA dosing strategies. Future studies should investigate optimal TXA dosing and administration methods to enhance bleeding management in staged bilateral TKA.

## Introduction

The management of bilateral knee osteoarthritis with total knee arthroplasty (TKA) presents a clinical dilemma: simultaneous versus staged bilateral procedures. Despite extensive debate, a consensus remains elusive, with Ritter MA et al. [1] advocating for individualized decision-making based on patient-specific factors. Simultaneous TKA, while offering potential benefits, is inherently associated with increased risks, as highlighted by Fabi DW et al. [2], Richardson MK et al. [3], Spicer E et al. [4], and Urban MK et al. [5]. To mitigate these risks, Gerner P et al. [6] demonstrated that stringent patient selection, focusing on younger, healthier individuals, can significantly reduce complication rates. Conversely, staged TKA remains the preferred approach for elderly patients and those with significant comorbidities [7]. A key advantage of staged procedures lies in the ability to leverage insights gained from the first-stage surgery to optimize the second. Prior studies have documented inter-leg differences in staged TKA, including variations in hospital stay [8], operative time [9], postoperative analgesic requirements [10-12], and clinical outcomes [13].

Minimizing allogeneic blood transfusion in TKA remains a cornerstone of perioperative care. Recent evidence has highlighted the efficacy of tranexamic acid (TXA) in reducing blood loss and transfusion rates, leading to its endorsement by major orthopedic societies, including the American Academy of Orthopaedic Surgeons (AAOS) [14]. Sawant S et al.'s review [15] further supports TXA’s role in blood conservation during TKA, emphasizing ongoing research to refine its clinical application. In line with this trend, a protocol of single-dose TXA (1g) administration immediately prior to tourniquet release was implemented in March 2012. This study compared outcomes between patients receiving TXA and those who did not, demonstrating a significant reduction in blood loss and transfusion requirements in the TXA group [16].

This study presents a retrospective analysis of patients undergoing staged bilateral TKA. Patients were categorized into three groups based on TXA administration: those receiving no TXA, those receiving TXA only during the second-stage procedure, and those receiving TXA during both procedures. The primary objectives were to evaluate the predictive capacity of first-stage blood loss for second-stage blood loss and to assess the impact of varying TXA administration timing on hemostatic efficacy within the same patient. The clinical significance of this research lies in its potential to refine blood management strategies in staged bilateral TKA, ultimately enhancing patient safety.

## Materials and methods

The institutional review board of Healthcare Corporation Ashinokai, Gyoda, Saitama, Japan (ID number: 2024-1) approved the present study. All patients signed a consent form that included a description of the protocol and the potential complications of cement-related and TXA-related procedures. A total of 100 patients (200 knees) who underwent staged bilateral hybrid (cemented tibia, uncemented femur) primary TKA with the New Jersey LCS total knee system (DePuy, Warsaw, IN) between March 2011 and December 2024 were retrospectively recruited for the study. All patients had knee osteoarthritis. Patients chose which knee would undergo TKA first. The timing of the second TKA was also determined solely by the patient and depended on their perceived ability to tolerate additional pain and limitations to their activities of daily living during the postoperative period. Following advice from the ethics committee, the exclusion criteria were as follows: abnormal values of coagulation (platelet count < 8.0 x 10^4^/mL) and/or the fibrinolytic system (fibrinogen degradation products in plasma > 5 mg/L), peripheral vascular diseases, neurological problems, preoperative hemoglobin concentration < 10 g/dL, and a history of deep vein thrombosis.

Patients’ stratification

Since the administration of 1g TXA IV began in March 2012, 100 patients who underwent the same procedure between March 2002 and December 2024 were stratified into three groups based on their TXA administration status (Table 1).

**Table 1:** Patient stratification based on TXA usage. TXA: Tranexamic acid.

Group	Patients (knees)	Term	Details
Bilateral TXA non-use group (NT group)	53 patients, 106 knees	from March 2002 to February 2012	TXA non-use for both knees
Hybrid group (H group)	21 patients, 42 knees	1st side: before February 2012 and 2nd side: after March 2012	1st side: TXA non-use 2nd side: TXA use
Bilateral TXA use group (T group)	26 patients, 52 knees	From March 2012 onwards	TXA use for both knees

Surgical technique and postoperative protocol

All surgeries were performed by the same surgical team under general anesthesia. An MT-720 tourniquet system (Mizuho-Ika, Tokyo, Japan) was used, and the tourniquet was released after wound closure in all cases.

One surgeon performed all the TKAs using a standardized technique. In all cases, intramedullary femoral and extramedullary tibial resection guides were used. Each hole created in the distal femur for the intramedullary guides was filled with an autologous bone plug. Patients in the TXA administration group received 1000 mg of TXA (Transamin Injection 1000 mg/10mL; Daiichi-Sankyo Co Ltd, Japan) intravenously shortly before deflation of the tourniquet. Lateral retinacular release and patella replacement were not performed. Just after deflating the tourniquet, 400 mL of autologous blood, which was collected and preserved eight days before the surgery, was returned to the patient. A bulky Jones dressing with a foot pump (A-V Impulse System; Novamedix, Andover, UK) was applied to prevent deep vein thrombosis (DVT) in the knee extended position. The drains were routinely removed during the first dressing change on the first postoperative day. All patients received prophylactic perioperative antibiotics and analgesics for pain management. Regarding perioperative anticoagulant use, patients who were taking anticoagulants preoperatively discontinued their medication from one week before surgery until three days postoperatively. No patients were newly prescribed anticoagulants postoperatively. Furthermore, there was no difference in anticoagulant usage between the initial and subsequent surgeries. Patients were allowed full weight-bearing ambulation after drain removal.

Calculation of calculated blood loss (CBL)

CBL was calculated as described by Gross JB [17], using the change in hematocrit (Hct) from before surgery (Hctpre) to one week after surgery (Hctpost 1w), when the lowest Hct value was detected [18], as follows:

where Hctave was calculated as the average of Hctpre and Hctpost 1w.

PBV was normalized to the patient’s weight and height according to the Nadler formula [19] as follows: PBV= (k1 × height (m))^3^ + k2​× weight (kg)+ k3​ where (k1= 0.3669), (k2= 0.03219), and (k3= 0.6041) for men, and (k1= 0.3561), (k2= 0.03308), and (k3 = 0.1833) for women.

Statistical analyses

Normality assumptions were rejected based on the Q-Q plot, Kolmogorov-Smirnov test, and Shapiro-Wilk test. For comparisons of continuous variables, Wilcoxon's signed-rank test was used for paired data. Spearman’s rank correlation coefficient was employed to assess relationships between two variables. Correlation strengths were categorized as follows: strong (0.70-1.0), moderate (0.40-0.69), and weak (0.20-0.39).

Post-hoc power analyses were performed based on the present sample size with an alpha of 0.05. To detect a difference with an alpha of 0.05 (two-sided) and a power of 80%, the required sample size for Wilcoxon’s signed-rank test with an effect size of 0.5 was estimated to be 35 per group. For Spearman’s rank correlation coefficient, detecting a significant correlation with an alpha of 0.05 (two-sided) and a power of 80% with an effect size of 0.5 required a sample size of 66 per group. Due to the small sample sizes of the H and T groups, a post-hoc power analysis was conducted, revealing a statistical power of 56.6% for Wilcoxon's signed-rank test and 36.3% for Spearman's rank correlation coefficient.

All statistical analyses were performed using R version 4.4.1 (R Foundation for Statistical Computing, Vienna, Austria) and G*Power version 3.1. Values are presented as medians (25th percentile, 75th percentile), and a p-value of <0.05 was considered statistically significant.

## Results

In the NT group without TXA administration to either leg (Table 2) [20], the blood loss in the first leg was 793 ml (627, 1009 ml), and in the second leg, it was 813 ml (609, 1126 ml). There was no significant difference in blood loss between the two legs (p=0.403). However, a positive moderate correlation was observed between blood loss in the first and second legs (p < 0.001, r=0.437).

**Table 2:** Patient demographics of the non-tranexamic acid use group. Values are expressed as either n (count) or as the median (25th percentile, 75th percentile). TKA: Total Knee Arthroplasty; HSS score: Hospital for Special Surgery Score [20].

Parameter	1st TKA	2nd TKA
N= 53 (Male; N= 6/ Female; N= 47)		
Age (years)	72 (68, 77)	74 (70, 78)
Height (cm)	150 (148, 154)	151 (145, 154)
Weight (kg)	61 (54, 66)	59 (54, 67)
BMI (kg/m²)	27 (24, 29)	27 (24, 29)
Preoperative RBC (x10⁴/ml)	424 (399, 442)	418 (398, 448)
Postoperative RBC (x10⁴/ml)	328 (301, 353)	325 (292, 359)
Preoperative hematocrit (%)	39.7 (37.9, 43.4)	39.6 (38.0, 42.4)
Postoperative hematocrit (%)	31.5 (29.7, 33.8)	31.0 (27.7, 34.0)
HSS Score	41 (35, 50)	47 (39, 52)
Operation time (min.)	54 (49, 62)	51 (48, 59)
Tourniquet time (min.)	59 (53, 67)	57 (51, 61)
Interval between 1st and 2nd TKA (months)	11 (6, 21)	

In the hybrid group, where TXA was administered to the second leg but not the first (Table 3) [20], the blood loss in the TXA-naïve first leg was 940 ml (840, 1313 ml), while the TXA-treated second leg showed a blood loss of 894 ml (718, 1047 ml). Although there was a trend towards reduced blood loss with TXA, the difference did not reach statistical significance (p=0.070). Furthermore, no correlation was found between blood loss in the two legs (p=0.340).

**Table 3:** Patient demographics of the hybrid group. Values are expressed as either count (n) or median (25th percentile, 75th percentile). TKA: Total Knee Arthroplasty; HSS score: Hospital for Special Surgery score [20].

Parameter	1st TKA	2nd TKA
N = 21 (Male: N = 2 / Female: N = 19)		
Age (years)	70 (65, 73)	77 (72, 78)
Height (cm)	152 (147, 155)	149 (145, 155)
Weight (kg)	59 (55, 65)	62 (51, 66)
Body Mass Index (kg/m²)	26 (25, 24) *	27 (25, 28)
Preoperative red blood cell (x10⁴/ml)	441 (420, 455)	434 (420, 462)
Postoperative red blood cell (x10⁴/ml)	337 (321, 359)	344 (311, 376)
Preoperative hematocrit (%)	40.4 (39.3, 41.4)	41.9 (38.6, 43.6)
Postoperative hematocrit (%)	32.1 (29.9, 32.7)	33.0 (29.9, 35.6)
HSS Score	48 (36, 51)	46 (39, 51)
Operation time (min.)	59 (49, 63)	56 (54, 63)
Tourniquet time (min.)	62 (53, 67)	58 (56, 65)
Interval between 1st and 2nd TKA (months)	66 (37, 108)	

In the T group where both legs received TXA (Table 4) [20], the blood loss in the first leg was 718 ml (594, 865 ml), and in the second leg, it was 699 ml (553, 1024 ml). No significant difference in blood loss was observed between the two legs (p=0.671), and likewise, no correlation was found between blood loss in the two legs (p=0.074).

**Table 4:** Patient demographics of the tranexamic acid use group. Values are expressed as counts (n) or medians (25th percentile, 75th percentile). TKA: Total Knee Arthroplasty; HSS score: Hospital for Special Surgery score [20].

Parameter	1st TKA	2nd TKA
N = 26 (Male: N = 4 / Female: N = 22)		
Age (years)	71 (69, 77)	75 (71, 79)
Height (cm)	151 (146, 155)	152 (146, 155)
Weight (kg)	61 (55, 71)	61 (55, 73)
BMI (kg/m²)	27 (24, 29)	27 (24, 32)
Preoperative RBC (x10⁴/ml)	437 (406, 454)	419 (410, 441)
Postoperative RBC (x10⁴/ml)	339 (329, 377)	346 (328, 379)
Preoperative hematocrit (%)	40.4 (38.8, 41.9)	40.3 (38.7, 41.3)
Postoperative hematocrit (%)	31.8 (30.5, 34.7)	33.4 (30.5, 35.1)
HSS Score	47 (38, 55)	46 (33, 52)
Operation time (min.)	60 (56, 68)	58 (53, 64)
Tourniquet time (min.)	62 (59, 70)	62 (55, 66)
Interval between 1st and 2nd TKA (months)	15 (11, 41)	

## Discussion

This study provides a novel perspective on two critical challenges in staged bilateral TKA: predicting bleeding and understanding the variability in TXA efficacy.

In the NT group, it was observed that the bleeding volume of the second knee could be predicted from that of the first. This finding, derived from a within-subject comparative study minimizing inter-surgeon and inter-patient bias, supports the potential for 'tailored perioperative management based on bleeding prediction.' Specifically, the first knee's bleeding volume may serve as a valuable indicator to optimize blood transfusion preparation and hemostatic measures for the contralateral knee.

Conversely, the analysis of the TXA group revealed no significant correlation between the bleeding amounts of the first and second surgeries, which was initially expected. This suggests considerable inter-patient variability in TXA efficacy, indicating that a uniform TXA dosage may not be optimal for all patients. Based on previous studies [16, 21] demonstrating that 1g of IV TXA effectively reduces inter-subject bleeding, and assuming consistent intra-subject efficacy, it was anticipated that the hybrid group would exhibit a significant reduction in bleeding in the second knee, and the bilateral group would exhibit a significant positive correlation between bleeding volumes in both knees. However, the findings contradicted these expectations, emphasizing the necessity for individualized TXA dosing to maximize efficacy. Given the established minimum effective dose of 10 mg/kg [22] and a half-life of 2 hours [23], future prospective studies should investigate the impact of individual variations in blood concentration per body weight, intraoperative hemodynamics, and patient comorbidities on TXA efficacy. Our prior study [24] demonstrated that in TKA patients receiving a single 1g dose of TXA, visible blood loss was inversely correlated with hidden blood loss, while hidden blood loss was positively correlated with body weight. This strongly suggests that TXA's serum concentration is weight-dependent and influenced by perioperative hemodynamics.

In summary, this study suggests a new direction for bleeding management in staged bilateral TKA. Firstly, in perioperative management based on bleeding prediction, the bleeding volume of the second knee can be predicted from that of the first knee in the non-TXA group, potentially optimizing blood transfusion preparation and hemostatic measures. This is expected to reduce perioperative transfusion risk and improve patient safety. Secondly, regarding individualized TXA dosing, since TXA efficacy varies among individuals, individualized dosing, considering patient background and intraoperative hemodynamics, rather than uniform dosing, is desirable. Future studies clarifying the optimal TXA dose and administration method will enable more effective bleeding management.

Acknowledging the limitations of this single-center, retrospective analysis with a limited sample size, multi-center prospective studies are warranted to validate the findings. Consequently, further research is essential to determine optimal TXA administration protocols, including dosage and routes such as intravenous and local application at the surgical site, given the variability in current practices reported in recent studies [25-27]. Given that the institution employs preoperative autologous blood donation and postoperative salvaged blood reinfusion to minimize allogeneic transfusions, external validation in centers without these measures is also necessary.

## Conclusions

This study presents a novel approach to bleeding management in staged bilateral TKA. In patients not receiving TXA, we demonstrated the potential to predict second-knee bleeding from first-knee blood loss, thereby facilitating tailored perioperative care. Conversely, significant inter-individual variability in TXA efficacy was observed, emphasizing the necessity for individualized dosing strategies. The within-subject comparative design minimized bias, thereby strengthening the reliability of our bleeding prediction findings. However, the retrospective, single-center nature and the limited sample size constrain the generalizability of these results. Therefore, future multi-center, prospective studies are warranted to validate our findings and establish optimized TXA administration protocols.
